# Taxonomic Positions of a Nyuzenamide-Producer and Its Closely Related Strains

**DOI:** 10.3390/microorganisms10020349

**Published:** 2022-02-02

**Authors:** Hisayuki Komaki, Yasuhiro Igarashi, Tomohiko Tamura

**Affiliations:** 1 Biological Resource Center, National Institute of Technology and Evaluation (NBRC), Chiba 292-0818, Japan; tamura-tomohiko@nite.go.jp; 2 Biotechnology Research Center and Department of Biotechnology, Toyama Prefectural University, Toyama 939-0398, Japan; yas@pu-toyama.ac.jp

**Keywords:** biosynthetic gene, classification, nonribosomal peptide, PKS, *Streptomyces*, subspecies

## Abstract

*Streptomyces* sp. N11-34 is a producer of bicyclic peptides named nyuzenamides A and B. We elucidated its taxonomic position and surveyed its nonribosomal peptide synthetase (NRPS) and polyketide synthase (PKS) gene clusters by whole genome analysis. *Streptomyces* sp. N11-34 showed 16S rRNA gene sequence similarities of 99.9% and 99.8% to *Streptomyces hygroscopicus* NBRC 13472^T^ and *Streptomyces demainii* NRRL B-1478^T^, respectively. Although these members formed a clade in a phylogenetic tree based on 16S rRNA gene sequences, the clade split into two closely related subclades in multilocus sequence analysis (MLSA). One included *Streptomyces* sp. N11-34, *S. demainii* NRRL B-1478^T^, *S. hygroscopicus* NBRC 100766, *S. hygroscopicus* NBRC 16556 and *S. hygroscopicus* TP-A0867 and the other comprised *S. hygroscopicus* NBRC 13472^T^ and *S. hygroscopicus* NBRC 12859. These phylogenetic relationships were supported by phylogenomic analysis. Although *Streptomyces* sp. N11-34 was classified to *S. hygroscopicus* at the species level based on MLSA evolutionary distances and DNA–DNA relatedness, these distances and relatedness of members between the two subclades were comparatively far (0.004–0.006) and low (75.4–76.4%), respectively. *Streptomyces* sp. N11-34 possessed six NRPS, seven PKS and four hybrid PKS/NRPS gene clusters in the genome. Among the seventeen, ten were identified to be biosynthetic gene clusters (BGCs) of nyuzenamide, echoside, coelichelin, geldanamycin, mediomycin, nigericin, azalomycin, spore pigment, alchivemycin and totopotensamide, whereas the remaining seven were orphan in our bioinformatic analysis. All seventeen are conserved in *S. hygroscopicus* NBRC 100766, *S. hygroscopicus* NBRC 16556 and *S. hygroscopicus* TP-A0867. In contrast, *S. hygroscopicus* NBRC 13472^T^ and *S. hygroscopicus* NBRC 12859 lacked the BGCs of alchivemycin, totopotensamide, a nonribosomal peptide and a hybrid polyketide/nonribosomal peptide compound. This difference was in a good accordance with the abovementioned phylogenetic relationship. Based on phenotypic differences in addition to phylogenetic relationship, DNA–DNA relatedness and BGCs, strains of *S. hygroscopicus* should be reclassified to two subspecies: *S. hygroscopicus* subsp. *hygroscopicus* and a new subspecies, for which we proposed *S. hygroscopicus* subsp. *sporocinereus* subsp. nov. The type strain is NBRC 100766^T^ (=ATCC 43692^T^ = DSM 41460^T^ = INMI 32^T^ = JCM 9093^T^ = NRRL B-16376^T^ = VKM Ac-312^T^). *S. demainii* was classified in this subspecies.

## 1. Introduction

Nonribosomal peptides and polyketides are the two largest families in the secondary metabolites of actinomycetes. These compounds are structurally diverse and often exhibit pharmaceutically useful biological activities. Half to two thirds of the secondary metabolite–biosynthetic gene clusters (smBGCs) in each actinomycetal genome are nonribosomal peptide synthetase (NRPS), polyketide synthase (PKS) and hybrid PKS/NRPS gene clusters, while each strain, such as that of the genus *Streptomyces*, harbors dozen of smBGCs [1]. NRPS and PKS pathways share a similar biosynthetic mechanism. Backbones of these products are synthesized by incorporation of building blocks, such as amino acid or acyl-CoA, respectively, into the growing chains. Biosyntheses by NRPS and type-I PKS pathways are catalyzed by large modular enzymes with multiple domains, according to the co-linearity rule of assembly line fashion. A minimum NRPS module consists of an adenylation (A) domain for selecting the incoming amino acid, a condensation (C) domain for condensing the building block with the peptidyl intermediate from the previous module and a thiolation (T) domain for carrying the growing polypeptide chain. Similarly, a minimal PKS module consists of an acyltransferase (AT) domain for selecting incoming acyl-CoAs, a ketosynthase (KS) domain for condensing the new building block with the acyl intermediate from the previous module and an acyl carrier protein (ACP) domain for carrying the growing polyketide chain. Individual modules are responsible for the incorporation of either one amino acid or acyl-CoA as a building block into the chain [2,3]. Optional domains may be present in each module, which methylate or epimerize incorporated amino acid residues in nonribosomal peptides or reduce a keto group in polyketide chains. Thus, we can predict backbones of the products based on module numbers, domain organization and the substrates of A and AT domains in each gene cluster by bioinformatic analysis [3,4]. Hence, PKS and NRPS gene clusters are often investigated to access the potential of each strain to produce diverse secondary metabolites [5,6,7,8].

**Figure 1:**
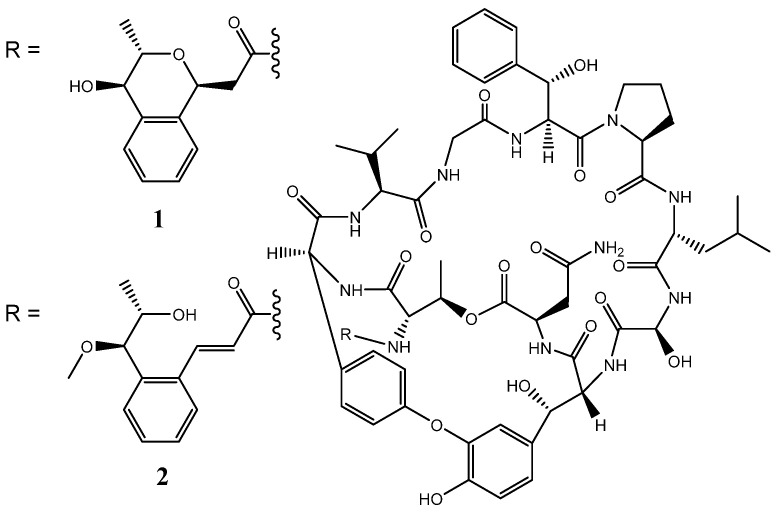
Chemical structures of nyuzenamides A (**1**) and B (**2**).

We recently isolated *Streptomyces* sp. N11-34 from deep sea water and found two novel compounds designated nyuzenamides A and B from the strain. Nyuzenamides are bicyclic peptides (Figure 1) with antifungal and cytotoxic activity [9]. Although these compounds seem to be synthesized through an NRPS pathway, the biosynthetic gene cluster (BGC) has not yet been elucidated. In the present study, we investigated the taxonomic position of *Streptomyces* sp. N11-34 and analyzed NRPS and PKS gene clusters to identify the nyuzenamide-BGC and reveal hidden potential to produce other compounds. Consequently, we classified *Streptomyces* sp. N11-34 to *S. hygroscopicus*.

*S.hygroscopicus* is known to include strains significant in industrial and biotechnological applications. As various bioactive secondary metabolites have been discovered from the members, it is expected as a source for searching novel bioactive compounds in pharmaceutical industries. This species once included four subspecies with validly published names. However, as they were reclassified to independent species, *S. hygroscopicus* includes no subspecies at present [10]. In the present study, we compared *S. hygroscopicus* N11-34 with its taxonomic neighbors, and consequently revealed that members of *S. hygroscopicus* can be classified into two groups. Thus, we here propose a new subspecies of *S. hygroscopicus*.

## 2. Materials and Methods

*Streptomyces* sp. N11-34 was isolated in the previous study [9]. This strain has been deposited to and available from the NBRC Culture Collection as NBRC 113678. EzBioCloud [11] was used to search for taxonomic neighbors based on 16S rRNA gene sequences. Multilocus sequence analysis (MLSA) was conducted using DNA sequences of five housekeeping genes—*atpD*, *gyrB*, *recA*, *rpoB* and *trpB*—as established in the genus *Streptomyces* [12]. The accession numbers of gene sequences used for MLSA are listed in Table S1. The phylogenetic trees were reconstructed using ClustalX 2.1 [13]. Genomic DNA of *Streptomyces* sp. N11-34 for whole genome sequencing was prepared from cultured cells via the method of Saito and Kimura [14]. The whole genome was sequenced by the Kazusa DNA Research Institute using a single-molecule real-time (SMRT) strategy in the same manner of our previous report [7]. The assembled genome sequences were deposited to DDBJ under the accession numbers BNEK01000001–BNEK01000009. Phylogenomic tree was constructed using the TYGS webserver [15]. DNA–DNA relatedness was digitally calculated using whole genome sequences by Formula 2 of the Genome-to-Genome Distance Calculator (GGDC), an in silico method that reliably mimics conventional DNA–DNA hybridization experiments [16]. PKS and NRPS gene clusters in the genomes were surveyed using antiSMASH, which allows the rapid genome-wide identification, annotation and analysis of smBGCs in microbial genomes [4], and then manually analyzed as reported previously [6]. Whole genome sequences used for DNA–DNA relatedness calculation and NRPS and PKS gene cluster analysis are listed in Table 1.

## 3. Results

### 3.1. Taxonomic Positions of Streptomyces sp. N11-34

*Streptomyces* sp. N11-34 showed 16S rRNA gene sequence similarities of 99.9% (1448/1449) and 99.8% (1446/1449) to *Streptomyces hygroscopicus* NBRC 13472^T^ and *Streptomyces demainii* NRRL B-1478^T^, respectively. In a phylogenetic tree based on 16S rRNA gene sequences, *Streptomyces* sp. N11-34 formed a clade with these members (Figure S1). As *S. hygroscopicus* has two heterotypic synonyms, *Streptomyces endus* and *Streptomyces sporocinereus* [17], we included their type strains in the tree. *S. hygroscopicus* TP-A0867 is an alchivemycin producer [18]. *S. hygroscopicus* NBRC 16556 is a strain for which we reported the whole genome sequence [19].

**Figure 2:**
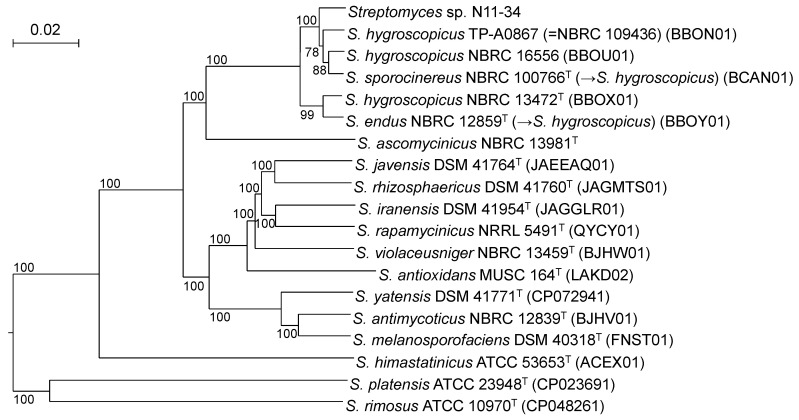
Phylogenomic tree constructed by the TYGS server. Confidence limits above 50% are at branching points. The codes in parentheses show accession numbers of whole genome sequences or WGS Projects. *S. albus* NBRC 13014^T^ (BBQG01) was used as an outgroup (not shown).

As 16S rRNA gene sequence analysis is known to be low in the resolution, we next conducted MLSA and phylogenomic analysis. MLSA is often used for elucidating phylogenetic relationships with higher resolutions [12], whereas phylogenomic analysis can clarify whole genome sequence-based phylogenies [15]. In the MLSA-based phylogenetic tree, *Streptomyces* sp. N11-34 formed a clade with members of *S. hygroscopicus* and *S. demainii*. However, the topology within the clade was different from that in the 16S rRNA gene sequence-based phylogenetic tree. The clade clearly split into two subclades with the bootstrap values of 100%: one comprises only the type strains of *S. hygroscopicus* and *S. endus* whereas the other is composed of the remaining members, including *Streptomyces* sp. N11-34 and the type strains of *S. sporocinereus* and *S. demainii* (Figure S2). Similarly, the members within the *S. hygroscopicus* clades split into two in the phylogenomic tree (Figure 2).

DNA–DNA relatedness value of 70% is established as the cut-off for species delineations in bacteria systematics [20]. In the genus *Streptomyces*, 0.007 in MLSA evolutionary distance is recognized to correspond to the cut-off [12]. Among *Streptomyces* sp. N11-34 and the phylogenetic neighbors, the DNA–DNA relatedness and MLSA evolutionary distances ranged 75.4–90.1% and 0.000–0.006, respectively (Table 2). This suggests that these members represent the same species. Hence, *Streptomyces* sp. N11-34 is classified to *S. hygroscopicus*. The seven strains have been phylogenetically grouped into two, one is **1**–**5** and the other is **6**–**7**, as stated above. Within the group **1**–**5**, DNA–DNA relatedness and MLSA evolutionary distances are 85.0–91.8% and 0.000–0.003, respectively. Similarly, these values are 90.1% and 0.000 within the other **6**–**7**, respectively. In contrast, values between the two groups are 75.4–76.4% and 0.004–0.006, respectively. As the threshold for subspecies demarcation is reported to be 79–80% in the DNA–DNA relatedness in bacteria [21], members between groups **1**–**5** and **6**–**7** are discriminated at subspecies level.

### 3.2. NRPS and PKS Gene Clusters of Streptomyces sp. N11-34

*Streptomyces* sp. N11-34 harbored six NRPS, seven PKS and four hybrid PKS/NRPS gene clusters in the genome, as listed in Table 3. We identified *nrps-1* and *-2* to be BGCs for echoside and coelichelin, respectively, by bioinformatic analysis. The NRPSs showed high similarities to EchA and SCO0492, responsible for echoside and coelichelin biosyntheses, respectively (Table 4). Although *nrps-3* was not a reported gene cluster, we identified it to be the nyuzenamide-BGC because the domain organization well accounts for it. Predicted amino acid residues of the A domains (Thr–X–Val–Gly–Phe–Pro–Leu–Gly–Tyr–Asn) are in a good accordance with those in nyuzenamides (Thr–Hpg–Val–Gly–Hpa–Pro–Leu–Hgy–Htr–Asn). As *nrps-4*, *-5* and *-6* were also unreported gene clusters, we predicted their products to be an octapeptide derived from dX–Thr–dX–Val–dX–dVal–dAla–Val, a Thr-containing molecule and a tripeptide including Gly, respectively, based on the domain organization and predicted substrate of A domains. Among the seven PKS gene clusters in this strain, four type-I PKS (*t1pks*) and one type-II (*t2pks*) gene clusters were identified to be BGCs of geldanamycin, mediomycin, nigericin, azalomycin and spore pigment, respectively, which were supported by high sequence similarities of the NRPSs and PKSs to the reported enzymes (Table 4). The remaining two were not reported gene clusters. Domain organization of *t1pks-5* partially resembled that of a butylolactol-BGC. Although the butyrolactol-BGC encodes six PKSs [22], *t1pks-5* encodes only five of the six. As the module number of *t1pks-5* is eight, the product was predicted to be a compound derived from an octaketide, which is similar to butyrolactol, but the alkyl chain is shorter than that of butyrolactol. Only a single module was present in *t1pks-6*. As it did not show similarities to BGCs of known compounds, the product could not be predicted. Among the hybrid PKS/NRPS gene clusters in this strain, *pks/nrps-1* and *-2* were BGCs of alchivemycin [18] and totopotensamide, respectively, whose NRPSs and PKSs correspond to the biosynthetic enzymes (Table 4). In contrast, *pks/nrps-3* and *-4* were orphan gene clusters. PKSs in *pks/nrps-3* were AT-less and resembled to those of leinamycin. However, as the domain organization differed from that of leinamycin-BGC, the product was predicted to be a new macrolactam compound like leinamycin. According to module number and domain organization, *pks/nrps-4* was predicted to synthesis octapeptide with a polyketide moiety.

### 3.3. Distributions of the NRPS and PKS Gene Clusters in Streptomyces sp. N11-34 to the Phylogenetically Close Strains

As *S. hygroscopicus* NBRC 100766, NBRC 16556, TP-A0867, NBRC 12859 and NBRC 13472^T^ are phylogenetically close to *Streptomyces* sp. N11-34, as described in the Section 3.1, we examined whether the seventeen PKS and NRPS gene clusters found in *Streptomyces* sp. N11-34 are present in the genomes of these *S. hygroscopicus* strains. As summarized in Table 5, all the gene clusters were present in *S. hygroscopicus* NBRC 100766, NBRC 16556 and TP-A0867, which are closer to *Streptomyces* sp. N11-34, but *S. hygroscopicus* NBRC 12859 and NBRC 13472^T^, phylogenetically discriminated from *Streptomyces* sp. N11-34, lacked *nrps-6, pks/nrps-1* (*avm*), *-2* (*tot*) and *-3*.

## 4. Discussion

The description of *S. hygroscopicus* (Jensen 1931) Yüntsen et al. 1956 (Approved Lists 1980) was emended in 2017, and this species has two heterotypic synonyms, *S. endus* Anderson and Gottlieb 1952 (Approved Lists 1980) and *S. sporocinereus* (ex Krassilnikov 1970) Preobrazhenskaya 1986 [17]. Once, *S. hygroscopicus* had four subspecies with validly approved names, such as *S. hygroscopicus* subsp. *angustmyceticus*, *S. hygroscopicus* subsp. *decoyicus*, *S. hygroscopicus* subsp. *glebosus* and *S. hygroscopicus* subsp. *ossamyceticus*. However, these subspecies have been reclassified as independent species by rank up [10,12,23] or reclassified to a synonym of another species [24]. Consequently, *S. hygroscopicus* has no subspecies at present [10]. In the present study, *Streptomyces* sp. N11-34 was classified to *S. hygroscopicus* as well as *Streptomyces* sp. TP-A0867, an alchivemycin producer [18], and *S. hygroscopicus* NBRC 16556 [19]. We have proposed a hypothesis that strains classified to the same species harbor a similar set of NRPS and PKS gene clusters in the genomes [6,7]. Although our present study supported the hypothesis in principle, it was unexpectedly observed that *S. hygroscopicus* NBRC 13472^T^ and NBRC 12859 lack four NRPS and PKS gene clusters among the seventeen clusters present in *Streptomyces* sp. N11-34. The lack was well correlated with the phylogenetic relationship since *S. hygroscopicus* NBRC 13472^T^ and NBRC 12859 were phylogenetically discriminated from the other members examined here. As summarized in Table 6, many different features were observed between *Streptomyces* sp. N11-34, *S. demainii* DSM 41600^T^, *S. hygroscopicus* NBRC 107666, NBRC 16556 and TP-A 0867 (group A) and *S. hygroscopicus* NBRC 13472^T^ and NBRC 12859 (group B). Although whole genome sequence of *S. demainii* DSM 41600^T^ has not been published, genome sizes are larger in the group A. Housekeeping gene sequences differ between the two groups. Members of the group B lack four smBGCs in their genome. The distinctive phenotypic characteristics between the groups A and B are given in the previous reports as follows: spore wall ornamentations are warty or rugose in the group A whereas those are smooth in the group B [25]; although members of the group A utilize D-fructose as a sole carbon source for growth, those of the group B do not [25,26]; and maltose utilization is stronger in members of the group A than of the group B [12]. Taken together, it is considered that members in the group A are a new subspecies of *S. hygroscopicus*, for which we propose *Streptomyces hygroscopicus* subsp. *sporocinereus* subsp. nov.

## 5. Descriptions of *Streptomyces hygroscopicus* and Its Subspecies

### 5.1. Description of Streptomyces hygroscopicus subsp. sporocinereus subsp. nov.

*Streptomyces hygroscopicus* subsp. *sporocinereus* (spo.ro.ci.ne’re.us. Gr. n. *spora* seed; L. adj. *cinereus* ash-colored; N.L. masc. adj. *sporocinereus* ash-colored spores).

The description is as given for *Streptomyces sporocinereus* (ex Krassilnikov 1970) Preobrazhenskaya 1986 [25,27]. This subspecies is also discriminated from *Streptomyces hygroscopicus* subsp. *hygroscopicus* by the genomic feature shown in Table 5. The genome size ranges from 9.9–10.4 Mb. The type strain is NBRC 100766^T^ (=ATCC 43692^T^ = DSM 41460^T^ = INMI 32^T^ = JCM 9093^T^ = NRRL B-16376^T^ = VKM Ac-312^T^). Accession numbers of the 16S rRNA gene and whole genome sequences in the type strain are AB249933 and BCAN01000001–BCAN01000217, respectively.

*Streptomyces demainii* Goodfellow et al. 2008 is included in this subspecies. *Streptomyces sporocinereus* (ex Krassilnikov 1970) Preobrazhenskaya 1986 is a basonym of this subspecies.

### 5.2. Emended Description of Streptomyces hygroscopicus subsp. hygroscopicus (Jensen 1931) Yüntsen et al. 1956 (Approved Lists 1980) emend. Komaki et al. 2017

The description is as given for *Streptomyces hygroscopicus* subsp. *hygroscopicus* (Jensen 1931) Yüntsen et al. 1956 (Approved Lists 1980) emend. Komaki et al. 2017 [17] with the following modifications. The genome size of the type strain is 9.5 Mb. *Streptomyces sporocinereus* (ex Krassilnikov 1970) Preobrazhenskaya 1986 is not included in this subspecies. *Streptomyces endus* Anderson and Gottlieb 1952 (Approved Lists 1980) is a member of this subspecies.

### 5.3. Emended Description of Streptomyces hygroscopicus (Jensen 1931) Yüntsen et al. 1956 (Approved Lists 1980)

The description is as given for *Streptomyces hygroscopicus* subsp. *hygroscopicus* (Jensen 1931) Yüntsen et al. 1956 (Approved Lists 1980) emend. Komaki et al. 2017 [17] with the following modifications. Spore wall ornamentation is smooth, warty or rugose. Utilization of D-fructose is different between its subspecies. Genome sizes range from 9.5–10.4 Mb. Accession numbers of 16S rRNA gene and whole genome sequences in the type strain are AB184428 and BBOX01000001–BBOX01000680, respectively. *Streptomyces endus* Anderson and Gottlieb 1952 (Approved Lists 1980), *Streptomyces demainii* Goodfellow et al. 2008 and *Streptomyces sporocinereus* (ex Krassilnikov 1970) Preobrazhenskaya 1986 are later heterotypic synonyms of this species [12,17].

## Figures and Tables

**Table 1:** Whole genome sequences of *Streptomyces* sp. N11-34 and its taxonomic neighbors.

Strain	WGS pj. ^1^	Scaffolds ^2^	Genome Size	G + C Content	CDS ^3^
*Streptomyces* sp. N11-34	BNEK01	9	10.41 Mb	71.9%	8824 ^4^
*S. hygroscopicus* NBRC 13472^T^	BBOX01	680	9.46 Mb	72.0%	7475
*S. hygroscopicus* NBRC 12859	BBOY01	539	9.78 Mb	71.9%	7691
*S. hygroscopicus* NBRC 16556	BBOU01	133	10.14 Mb	72.0%	7887
*S. hygroscopicus* NBRC 100766	BCAN01	217	10.17 Mb	72.0%	7886
*S. hygroscopicus* TP-A0867	BBON01	259	9.89 Mb	72.0%	7584 ^5^

^1^ Prefix for accession number of whole genome shotgun (WGS) project; ^2^ number of scaffolds; ^3^ number of CDS, taken from https://www.ncbi.nlm.nih.gov/genome/browse/#!/prokaryotes/1889/ (accessed on 25 January 2022); ^4^ protein number from https://www.ncbi.nlm.nih.gov/Traces/wgs/BNEK01?display=proteins&page=1 (accessed on 25 January 2022); ^5^ from #577 on https://www.ncbi.nlm.nih.gov/genome/browse/#!/prokaryotes/13511/ (accessed on 25 January 2022).

**Table 2:** MLSA evolutionary distances and DNA–DNA relatedness among *Streptomyces* sp. N11-34 and the phylogenetically close strains.

Strain	DNA–DNA Relatedness (%)						
	1	2	3	4	5	6	7
**1**. *Streptomyces* sp. N11-34	-	85.1	nd ^2^	85.4	85.0	75.9	75.4
**2**. *S. sporocinereus*^1^ NBRC 100766^T^	0.002	-	nd ^2^	87.6	91.8	76.4	76.1
**3**. *S. demainii* DSM 41600^T^	0.002	0.002	-				
**4**. *S. hygroscopicus* TP-A0867	0.002	0.001	0.003	-	88.2	76.2	76.2
**5**. *S. hygroscopicus* NBRC 16556	0.002	0.000	0.002	0.001	-	76.3	76.2
**6**. *S. hygroscopicus* NBRC 13472^T^	0.006	0.004	0.005	0.006	0.004	-	90.1
**7**. *S. endus*^1^ NBRC12859^T^	0.006	0.004	0.005	0.006	0.004	0.000	-
	**MLSA Evolutionary Distance**						

^1^ The correct names are *S. hygroscopicus,* because these two species are reclassified to *S. hygroscopicus* [17]. ^2^ not determined.

**Table 3:** NRPSs and PKSs in these gene clusters in the genome of *Streptomyces* sp. N11-34.

Gene Cluster	ORF ^1^ (TPA0910_)	Domain Organization	Putative Product
NRPS			
*nrps-1* (*ech*)	57730	A/T-Te	Echoside
*nrps-2*	86030	A_orn_/T/E-C/A_thr_/T/E-C/A_orn_/T	Coelichelin
*nrps-3*	18360	C/A_phe_/T-C/A_pro_/T-C/A_leu_/T/E	Nyuzenamides
	18370	C/A_gly_/T-C/A_tyr_/T/E-C/A_asn_/T-Te	
	18410	C/A_thr_/T-C/A/T-C/A_val_/T-C/A_gly_/T/E	
*nrps-4*	6570	E-C/A_val_/T/E-C/A_ala_/T/E-C/A_val_/T	dX-Thr-dX-Val-dX-dVal-dAla-Val
	6550	A/T/E-C/A_thr_/T-C/A/T/E-C/A_val_/T-C/A/T/E	
*nrps-5*	23490	C/A_thr_/T-Te	Thr-containing molecule
*nrps-6*	40370	Te	Tripeptide including Gly
	40360	A_gly_/T-C/A/T	
	40350	A/T	
PKS			
*t1pks-1* (*gdm*)	01850	CoL/KR/ACP-KS/AT_mm_/DH/ER/KR/ACP	Geldanamycin
		-KS/AT_mx_/DH/ER/KR/ACP-KS/AT_mm_/KR/ACP	
	01840	KS/AT_mm_/DH/KR/ACP-KS/AT_mx_/KR/ACP	
	01830	KS/AT_m_/DH/ER/KR/ACP-KS/AT_mm_/DH/KR/ACP	
*t1pks-2* (*cle*)	26380	ACP-KS/AT_m_/KR/ACP-KS/AT_m_/KR/ACP	Mediomycin
		-KS/AT_m_/DH/KR/ACP-KS/AT_m_/KR/ACP	
		-KS/AT_m_/KR/ACP	
	26390	KS/AT_m_/DH/KR/ACP-KS/AT_m_/KR/ACP	
	26400	KS/AT_m_/DH/KR/ACP-KS/AT_m_/KR/ACP	
		-KS/AT_m_/DH/KR/ACP-KS/AT_m_/KR/ACP	
		-KS/AT_m_/DH/KR/ACP	
	26410	KS/AT_mm_/KR/ACP	
	26420	KS/AT_m_/KR/ACP-KS/AT_m_/DH/KR/ACP	
		-KS/AT_m_/DH/KR/ACP	
	26430	KS/AT_m_/DH/KR/ACP-KS/AT_m_/DH/KR/ACP	
		-KS/AT_m_/DH/KR/ACP-KS/AT_m_/DH/KR/ACP	
	26440	KS/AT_mm_/KR/ACP-KS/AT_mm_/KR/ACP	
	26450	KS/AT_m_/DH/KR/ACP-KS/AT_m_/DH/KR/ACP	
		-KS/AT_m_/DH/KR/ACP	
	26460	KS/AT_m_/DH/KR/ACP-KS/AT_mm_/DH/KR/ACP-Te	
*t1pks-3* (*nig*)	77850	KS/AT_m_/ACP-KS/AT_mm_/DH/ACP	Nigericin
	77860	KS/AT_mm_/DH/KR/ACP	
	77870	KS/AT_m_/DH/KR/ACP-KS/AT_mm_/DH/ER/KR/ACP	
	77880	KS/AT_mm_/DH/KR/ACP	
		-KS/AT_m_/DH/ER/KR/ACP	
	77890	KS/AT_mm_/DH/KR/ACP	
		-KS/AT_mm_/DH/ER/KR/ACP	
	77900	KS/AT_mm_/KR/ACP	
	77930 ^2^	ACP	
	77940 ^2^	KS/KR	
	77980 ^2^	KS/AT_mm_/DH/KR/ACP	
	77990 ^2^	KS/AT_mm_/DH/ER/KR/ACP	
	78000 ^2^	KS/AT_m_/KR/ACP-KS/AT_m_/KR/ACP	
		-KS/AT_m_/KR/ACP	
*t1pks-4* (*azl*)	79560	KS/AT_mm_/DH/KR/ACP-KS/AT/DH/ER/KR/ACP	Azalomycin
	79570	KS/AT/KR/ACP-KS/AT_m_/DH/KR/ACP	
	79580	KS/AT/KR/ACP	
	79590	KS/AT_m_/KR/ACP-KS/AT_m_/KR/ACP	
		-KS/AT_m_/KR/ACP	
	79600	KS/AT_m_/KR/ACP-KS/AT_m_/KR/ACP	
		-KS/AT_m_/DH/ACP-KS/AT/KR/ACP	
		-KS/AT_m_/DH/ER/KR/ACP	
	79610	KS/AT/KR/ACP-KS/AT_m_/KR/ACP	
	79620	KS/AT/KR/ACP-KS/AT_m_/DH/KR/ACP	
	79630	KS/AT/DH/KR/ACP-Te	
	79640	ACP-KS/AT_m_/DH/ER/KR/ACP	
*t1pks-5*	600	KS/AT_mm_/ACP-KS/AT_mm_/DH/ER/KR/ACP	Octaketide like butyrolactol
	610	KS/AT_mm_/DH/KR/ACP	
	620	KS/AT_mm_/DH/ER/KR/ACP-KS/AT/KR/ACP	
	630	KS/AT_m_/KR/ACP-KS/AT_m_/KR/ACP	
	640	KS/KR/ACP	
*t1pks-6*	41390	ACP-KS/AT/DH/KR	Unknown
	41400	Te	
*t2pks* (*spp*)	66950	KSα	Spore pigment
	66940	KSβ (CLF)	
	66930	ACP	
Hybrid PKS/NRPS			
*pks/nrps-1* (*avm*)	49610	C/A_gly_/T	Alchivemycin
	49640	KS/AT_m_/ACP-KS/AT_mm_/DH/KR/ACP	
	49650	KS/AT_m_/ACP-KS/AT_m_/KR/ACP	
	49660	KS/AT_mm_/KR/ACP-KS/AT_m_/DH/ER/KR/ACP	
	49670	KS/AT_m_/DH/ER/KR/ACP	
		-KS/AT_mm_/DH/KR/ACP	
		-KS/AT_m_/DH/KR/ACP-KS/AT_m_/DH/KR/ACP	
	49680	KS/AT_mm_/DH/ER/KR/ACP	
		-KS/AT_mm_/DH/ER/KR/ACP	
	49690	KS/AT_m_/DH/KR/ACP-KS/AT_m_/KR/ACP	
*pks/nrps-2* (*tot*)	85480	KS (type-III PKS)	Totopotensamide
	85540	KS/AT_m_/ACP-KS/AT_mm_/DH/KR/ACP	
		-KS/AT_mm_/KR/ACP	
		-KS/AT_mm_/DH/ER/KR/ACP	
	85550	KS/AT_mm_/DH/ER/KR/ACP-KS/AT_m_/KR/ACP	
	85650	C/A_thr_/T-C/A/T-C/T-C/A/T-C/A_val_/T-C/A/T-Te	
	86660	A/T	
	85750	C/T	
*pks/nrps-3*	65820	AT_m_	Macrolactam like leinamycin
	65840	A_thr_/T	
	65870	T/C-C/A_cys_/T-KS/ACP-KS/KR/ACP-KS	
	65880	DH/ACP/KR-KS/ACP/DH/ECH/ACP	
		-KS/KR/ACP-KS/ACP/ACP	
*pks/nrps-4*	77610	Te	pk-Thr-Phe-dX-Val-Pro-X-mTyr-Pro
	77590	KR	
	77530 ^2^	KS/AT_mm_/DH/ER/KR/ACP	
	77520 ^2^	KS	
	77490	A_thr_/T-C/A_phe_/T-C/A/T/E-C/A_val_/T-C/A_pro_/T	
		-C/A/T-C/A_tyl_/MT/T-C/A_pro_/T-Te	

^1^ shown by locus tag; ^2^ encoded in the complementary strand. Abbreviations are as follows: A, adenylation; ACP, acyl carrier protein; AT, acyltransferase; AT_m_, AT for malonyl-CoA; AT_mm_, AT for methyl malonyl-CoA; AT_mx_, AT for methoxymalonyl-CoA; C, condensation; CLF, chain length factor; CoL, CoA ligase; d, D-; DH, dehydratase; E, epimerization; ER, enoyl reductase; KR, ketoreductase; KS, ketosynthase; MT, methyltransferase; *nrps*, NRPS gene cluster; pk, polyketide; *pks/nrps*, hybrid PKS/NRPS gene cluster; T, thiolation; Te, thioesterase; *t1pks*, type-I PKS gene cluster; *t2pks*, type-II PKS gene cluster; X, unidentified amino acid residue; Amino acids incorporated by A domains are indicated as 3-letter abbreviations in subscript just after A.

**Table 4:** Similarities of enzymes involved in the biosynthesis of known compounds to those of *Streptomyces* sp. N11-34.

Gene Cluster (Putative Product)	ORF (TPA0910_) ^1^	I/S (%) ^2^	Known Biosynthetic Enzyme (Accession, Origin)
*nrps-1* (echoside)	57730	91/94	EchA (AHN91924, *Streptomyces* sp. LZ35)
*nrps-2* (coelichelin)	86030	81/86	SCO0492 (CAB53322, *S. coelicolor* A3(2)
*t1pks-1* (geldanamycin)	1850	84/88	GelA (ABB86408, *S. hygroscopicus* subsp. *duamyceticus*)
	1840	83/87	GelB (ABB86409, *S. hygroscopicus* subsp. *duamyceticus*)
	1830	85/89	GelC (ABB86410, *S. hygroscopicus* subsp. *duamyceticus*)
*t1pks-2* (mediomycin)	26380	76/82	Cle1 (AWC08655, *Kitasatospora mediocidica*)
	26390	77/83	Cle2 (AWC08656, *K. mediocidica*)
	26400	78/84	Cle3 (AWC08657, *K. mediocidica*)
	26410	80/87	Cle4 (AWC08658, *K. mediocidica*)
	26420	79/85	Cle5 (AWC08659, *K. mediocidica*)
	26430	79/85	Cle6 (AWC08660, *K. mediocidica*)
	26440	83/88	Cle7 (AWC08661, *K. mediocidica*)
	26450	81/87	Cle8 (AWC08662, *K. mediocidica*)
	26460	79/86	Cle9 (AWC08663, *K. mediocidica*)
*t1pks-3* (nigericin)	77850	83/87	NigAI (ABC84456, *S. violaceusniger*)
	77860	83/87	NigAII (ABC84457, *S. violaceusniger*)
	77870	85/88	NigAIII (ABC84458, *S. violaceusniger*)
	77880	81/86	NigAIV (ABC84459, *S. violaceusniger*)
	77890	81/85	NigAV (ABC84460, *S. violaceusniger*)
	77900	81/85	NigAVI (ABC84461, *S. violaceusniger*)
	77930	90/93	NigC1 (ABC84466, *S. violaceusniger*)
	77940	82/87	NigAX (ABC84465, *S. violaceusniger*)
	77980	81/85	NigAIX (ABC84469, *S. violaceusniger*)
	77990	84/88	NigAVIII (ABC84470, *S. violaceusniger*)
	78000	83/88	NigAVII (ABC84471, *S. violaceusniger*)
*t1pks-4* (azalomycin)	79560	83/88	AzlB (ARM20277, *Streptomyces* sp. 211726)
	79570	84/89	AzlB (ARM20277, *Streptomyces* sp. 211726)
	79580	63/73	AzlC (ARM20278, *Streptomyces* sp. 211726)
	79590	85/90	AzlD (ARM20279, *Streptomyces* sp. 211726)
	79600	90/94	AzlE (ARM20280, *Streptomyces* sp. 211726)
	79610	93/96	AzlF (ARM20281, *Streptomyces* sp. 211726)
	79620	75/83	AzlG (ARM20282, *Streptomyces* sp. 211726)
	79630	80/86	AzlH (ARM20283, *Streptomyces* sp. 211726)
	79640	92/95	AzlA (ARM20284, *Streptomyces* sp. 211726)
*t2pks* (spore pigment)	66950	77/86	SppA (BAC70549, *S. avermitilis*)
	66940	73/83	SppB (BAC70550, *S. avermitilis*)
	66930	54/71	SppC (BAC70551, *S. avermitilis*)
*pks/nrps-1* (alchivemycin)	49610	96/96	AvmN (QSV12656, *Streptomyces* sp. NBRC 109436)
	49640	95/96	AvmA (QSV12655, *Streptomyces* sp. NBRC 109436)
	49650	95/95	AvmB (QSV12659, *Streptomyces* sp. NBRC 109436)
	49660	97/97	AvmC (QSV12661, *Streptomyces* sp. NBRC 109436)
	49670	97/97	AvmD (QSV12662, *Streptomyces* sp. NBRC 109436)
	49680	97/97	AvmE (QSV12663, *Streptomyces* sp. NBRC 109436)
	49690	95/95	AvmF (QSV12664, *Streptomyces* sp. NBRC 109436)
*pks/nrps-2* (totopotensamide)	85480	81/87	TotC1 (ATL73040, *S. pactum*)
	85540	67/75	TotA2 (ATL73034, *S. pactum*)
	85550	68/75	TotA1 (ATL73033, *S. pactum*)
	85650	73/79	TotB1 (ATL73036, *S. pactum*)
	86660	76/82	TotB2 (ATL73045, *S. pactum*)
	85750	65/72	TotB3 (ATL73046, *S. pactum*)

^1^ shown by locus tag; ^2^ identity/similarity in amino acid sequences.

**Table 5:** Distribution of the NRPS and PKS gene clusters in *Streptomyces* sp. N11-34 to the phylogenetically close *S. hygroscopicus* strains.

Gene Cluster (Product)	N11-34	100766	16556	TP-A	12859	13472^T^
NRPS						
*nrps-1* (echoside)	+	+	+	+	+	+
*nrps-2* (coelichelin)	+	+	+	+	+	+
*nrps-3* (nyuzenamide)	+	+	+	+	+	+
*nrps-4*	+	+	+	+	+	+
*nrps-5*	+	+	+	+	+	+
*nrps-6*	+	+	+	+	−	−
PKS						
*t1pks-1* (geldanamycin)	+	+	+	+	+	+
*t1pks-2* (mediomycin)	+	+	+	+	+	+
*t1pks-3* (nigericin)	+	+	+	+	+	+
*t1pks-4* (azalomycin)	+	+	+	+	+	+
*t1pks-5*	+	+	+	+	+	+
*t1pks-6*	+	+	+	+	+	+
*t2pks* (spore pigment)	+	+	+	+	+	+
Hybrid PKS/NRPS						
*pks/nrps-1* (alchivemycin)	+	+	+	+	−	−
*pks/mrps-2* (totopotensamide)	+	+	+	+	−	−
*pks/nrps-3*	+	+	+	+	−	−
*pks/nrps-4*	+	+	+	+	+	+

+, present; −, absent; N11-34, *Streptomyces* sp. N11-34; 10766, *S. hygroscopicus* NBRC 100766 (type strain of *S. sporocinereus*); 16556, *S. hygroscopicus* NBRC 16556; TP-A, *S. hygroscopicus* TP-A0867; 12856, *S. hygroscopicus* NBRC 12856 (type strain of *S. endus*); 13472^T^, *S. hygroscopicus* NBRC 13472^T^. *S. sporocinereus* and *S. endus* are later heterotypic synonyms of *S. hygroscopicus.* Sequence similarities of PKS and NRPS genes in *Streptomyces* sp. N11-34 to those in the phylogenetically close *S. hygroscopicus* strains are shown in Table S2.

**Table 6:** Genotypic differentiation between the two groups.

Genotypic Feature	Group A						Group B	
	N11-34	*dema*	10766	16556	TP-A		12859	13472^T^
Genotypic								
Genome size (Mb)	10.4	nd	10.2	10.1	9.9		9.8	9.5
Different sequence in								
*atpD* (495 bp)	A^19^, T^53^, C^55^, G^80^, T^96^, C^142^, T^474^						T^19^, C^53^, A^55^, A^80^, C^96^, A^142^, G^474^	
*gyrB* (498 bp)	C^192^						T^192^	
*rpoB* (540 bp)	C^450^						T^450^	
*trpB* (567 bp)	C^96^						T^96^	
Biosynthetic gene cluster								
*nrps-6*	+	nd	+	+	+		−	−
*pks/nrps-1* (alchivemycin)	+	nd	+	+	+		−	−
*tot* (totopotensamide)	+	nd	+	+	+		−	−
*pks/nrps-3*	+	nd	+	+	+		−	−

+, present; −, lacked; N11-34, *Streptomyces* sp. N11-34; *dema*, *S. demainii* DSM 41600^T^; 10766, *S. sporocinereus* NBRC 100766^T^; 16556, *S. hygroscopicus* NBRC 16556; TP-A, *S. hygroscopicus* TP-A0867; 12859, *S. endus* NBRC 12859^T^; 13472^T^, *S. hygroscopicus* NBRC 13472^T^; nd, not determined. These strains are *S. hygroscopicus* at the species level.

## Data Availability

The whole genome shotgun project of *Streptomyces* sp. N11-34 has been deposited at DDBJ under the accession number BNEK00000000. Accession numbers of the BioProject and the BioSample are PRJDB9821 and SAMD00228011, respectively.

## Supplementary Materials

The following supporting information can be downloaded at: https://www.mdpi.com/article/10.3390/microorganisms10020349/s1, Figure S1: Phylogenetic tree based on 16S rRNA gene sequences, Figure S2: Phylogenetic tree based on MLSA, Table S1: Accession numbers of gene sequences used for MLSA.
