# Supplementary material for: Taxonomic Positions of a Nyuzenamide-Producer and Its Closely Related Strains
Source: Microorganisms. 2022 Feb 2;10(2):349. doi: 10.3390/microorganisms10020349 (PMC8880029; doi:10.3390/microorganisms10020349)
Supplement: Supplementary file 1 [file microorganisms-10-00349-s001.zip › microorganisms-1533197-supplementary.pdf]

microorganisms

Supplementary Materials of

Taxonomic Positions of a Nyuzenamide-Producer and Its Closely Related Strains

Hisayuki Komaki [1,*], Yasuhiro Igarashi [2] and Tomohiko Tamura [1]

[1] Biological Resource Center, National Institute of Technology and Evaluation (NBRC), Chiba 292-0818, Japan; tamura-tomohiko@nite.go.jp
[2] Biotechnology Research Center and Department of Biotechnology, Toyama Prefectural University, Toyama 939-0398, Japan; yas@pu-toyama.ac.jp
* Correspondence: komaki-hisayuki@nite.go.jp

Contents

Supplementary Figure S1
Phylogenetic tree based on 16S rRNA gene sequences page 2

Supplementary Figure S2
Phylogenetic tree based on MLSA page 3

Supplementary Table S1
Accession numbers of gene sequences used for MLSA page 4

Supplementary Table S2
Nucleotide sequence similarity of the NRPS and PKS genes in *Streptomyces* sp. N11-34 to those of the phylogenetically close *S. hygroscopicus* strains page 5

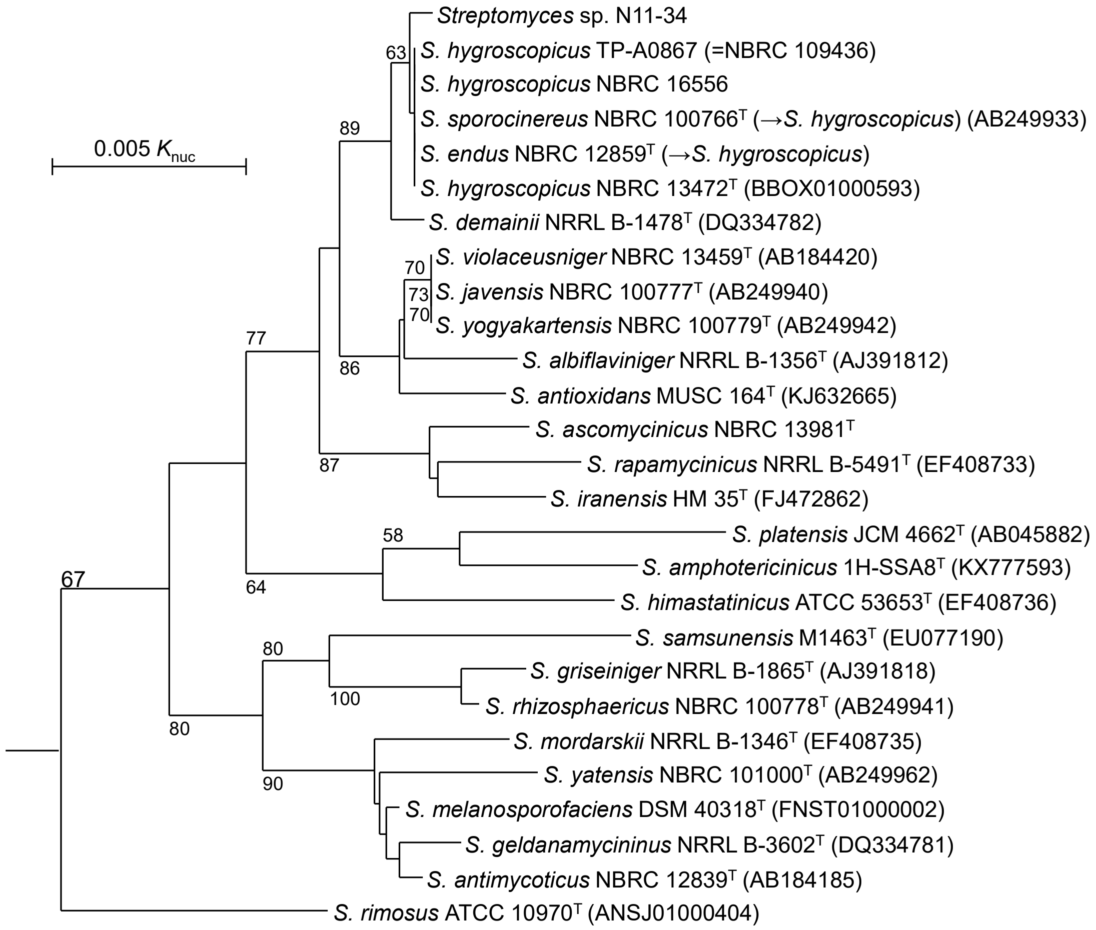

Figure S1. Phylogenetic tree based on 16S rRNA gene sequences. Numbers on the branches represent the confidence limits estimated by bootstrap analysis with 1,000 replicates; values above 50% are at branching points. The sequences of *Streptomyces* sp. N11-34, *S. hygroscopicus* strains TP-A0867, NBRC 16556, NBRC 12859, and *Streptomyces ascomycinicus* NBRC 13981ᵀ were from the NBRC Culture catalog (https://www.nite.go.jp/nbrc/catalogue/?lang=en). *Streptomyces albus* NBRC 13014ᵀ (AB184257) was used as an outgroup (not shown).

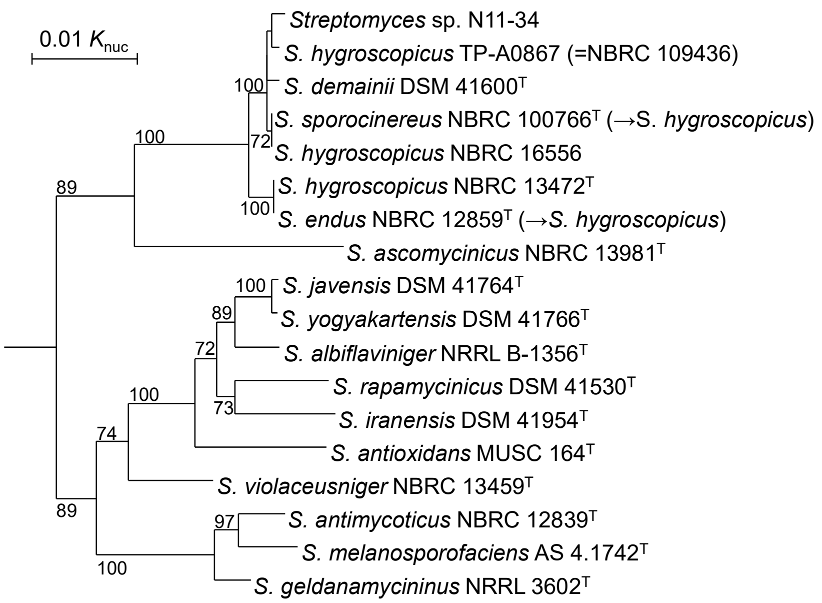

Figure S2. Phylogenetic tree based on MLSA. Numbers on the branches represent the confidence limits estimated by bootstrap analysis with 1,000 replicates; values above 50% are at branching points. *S. albus* NBRC 13014^{T} was used as an outgroup (not shown).

Table S1. Accession numbers of gene sequences used for MLSA.

Strain	*atpD*	*gyrB*	*recA*	*rpoB*	*trpB*
Streptomyces sp. N11-34	BNEK01000005	BNEK01000005	BNEK01000005	BNEK01000005	BNEK01000003
S. albiflaviniger NRRL B-1356^T	KT384458	KT384807	KT385155	KT388777	KT389127
S. albus NBRC 13014^T	BBQG01000033	BBQG01000007	BBQG01000035	BBQG01000012	BBQG01000017
S. antimycoticus NBRC 12839^T	BJHV01000001	BJHV01000001	BJHV01000001	BJHV01000001	BJHV01000001
S. antioxidans MUSC 164^T	LAKD02000030	LAKD02000040	LAKD02000035	LAKD02000005	LAKD02000088
S. demainii DSM 41600^T	FJ406182	FJ406238	FJ406294	FJ406349	FJ406405
S. geldanamycininus NRRL 3602^T	KT384563	KT384912	KT385261	KT388882	KT389232
S. javensis DSM 41764^T	JAEEAQ010000050	JAEEAQ010000059	JAEEAQ010000032	FJ406344	FJ406400
S. hygroscopicus NBRC 13472^T	BBOX01000262	BBOX01000097	BBOX01000200	BBOX01000138	BBOX01000120
S. hygroscopicus NBRC 12859	BBOY01000177	BBOY01000153	BBOY01000180	BBOY01000007	BBOY01000215
S. hygroscopicus NBRC 16556	BBOU01000024	BBOU01000028	BBOU01000034	BBOU01000036	BBOU01000075
S. hygroscopicus NBRC 100766	BCAN01000043	BCAN01000076	BCAN01000048	BCAN01000083	BCAN01000217
S. hygroscopicus TP-A0867	BBON01000044	BBON01000035	BBON01000036	BBON01000155	BBON01000092
S. iranensis DSM 41954^T	JAGGLR010000004	JAGGLR010000040	JAGGLR010000010	JAGGLR010000027	JAGGLR010000001
S. melanosporofaciens AS 4.1742^T	FJ406153	FJ406209	FJ406265	FJ406321	FJ406376
S. rapamycinicus DSM 41530^T	JACHNG010000001	JACHNG010000001	JACHNG010000001	JACHNG010000001	JACHNG010000001
S. violaceusniger NBRC 13459^T	BJHW01000001	BJHW01000001	BJHW01000001	BJHW01000001	BJHW01000001
S. yogyakartensis DSM 41766^T	FJ406178	FJ406234	FJ406290	FJ406345	FJ406401

Table S2. Nucleotide sequence similarity of the NRPS and PKS genes in *Streptomyces* sp. N11-34 to those of the phylogenetically close *S. hygroscopicus* strains.

Gene cluster (product)	Gene (locus tag)	Similarity to the gene of strain N11-34 (%)					
		N11-34	100766	16556	TP-A	12859	13472^T
nrps-1 (echoside)	TPA0910_57730	100	99.24	99.24	99.32	98.52	98.49
nrps-2 (coelichelin)	TPA0910_86030	100	99.22	99.22	99.31	98.52	98.58
nrps-3 (nyuzenamide)	TPA0910_18360	100	98.74	98.67	99.25	98.37*	98.18
	TPA0910_18370	100	99.71	99.73	99.24*	98.75	99.21
	TPA0910_18410	100	98.34	98.81	99.11	98.47	98.35
nrps-4	TPA0910_06570	100	99.37	99.37	99.03	98.99	98.90
	TPA0910_06550	100	99.35	99.49	99.40	99.02	98.98
nrps-5	TPA0910_23490	100	98.80	99.50	99.22	98.26	98.54
nrps-6	TPA0910_40370	100	99.86	99.86	99.59	no	no
	TPA0910_40360	100	99.29	99.35	99.47	no	no
	TPA0910_40350	100	99.39	99.36	99.54	no	no
t1pks-1 (geldanamycin)	TPA0910_01850	100	98.93	99.04	98.32	98.20	98.71
	TPA0910_01840	100	98.85	98.99	98.51	98.47	98.47
	TPA0910_01830	100	98.95	98.82	98.80	98.93	98.84
t1pks-2 (mediomycin)	TPA0910_26380	100	98.87	98.80	98.01	97.73	97.12
	TPA0910_26390	100	98.01	97.76	98.20	96.66	97.23
	TPA0910_26400	100	98.21	97.78	97.78	98.56	96.38
	TPA0910_26410	100	98.41	98.25	97.84	98.22	98.01
	TPA0910_26420	100	98.70	98.84	98.39	97.86	98.75
	TPA0910_26430	100	97.92	97.72	97.57	97.30	97.76
	TPA0910_26440	100	98.21	98.22	98.20	97.56	98.03
	TPA0910_26450	100	98.90	98.95	98.65	98.91	98.63
	TPA0910_26460	100	98.59	98.35	98.49	98.51	98.06
t1pks-3 (nigericin)	TPA0910_77850	100	99.47	99.13	98.67	98.45	99.26
	TPA0910_77860	100	95.68	95.88	97.35	97.82	98.40
	TPA0910_77870	100	98.66*	99.02	98.63	97.92	97.65
	TPA0910_77880	100	98.64	98.21	98.15	97.40*	96.91*
	TPA0910_77890	100	97.08	97.99*	97.82	96.75*	97.12*
	TPA0910_77900	100	98.72*	98.72*	98.69	96.02*	98.23*
	TPA0910_77930	100	98.82	98.82	99.12	99.12	99.41
	TPA0910_77940	100	99.14	98.66	97.85	97.10	96.96
	TPA0910_77980	100	99.24*	98.24*	98.88	98.13*	98.17*
	TPA0910_77990	100	99.44	99.71	99.40	98.40	98.50
	TPA0910_78000	100	98.42	98.72	98.45	98.00*	97.84*
t1pks-4 (azalomycin)	TPA0910_79560	100	90.40	90.38	92.86	98.17	98.11
	TPA0910_79570	100	99.14	99.14	99.18	92.22*	91.84*
	TPA0910_79580	100	99.68	99.76	99.41	86.32*	85.28*
	TPA0910_79590	100	99.58	99.62	99.64	87.10	87.63
	TPA0910_79600	100	99.69	99.64	99.75	87.80*	92.10*
	TPA0910_79610	100	99.13	99.47	99.15	89.68*	91.54*
	TPA0910_79620	100	99.47	99.43	99.55	91.06*	92.09*
	TPA0910_79630	100	99.89	99.86	99.92	88.45*	89.34*
	TPA0910_79640	100	99.48	99.51	99.46	98.62	98.68
t1pks-5	TPA0910_00600	100	99.39	99.30	99.39	98.48*	98.72*
	TPA0910_00610	100	97.07	97.13	96.70	97.68*	97.90
	TPA0910_00620	100	98.81	99.05	98.22	98.59	98.82*
	TPA0910_00630	100	99.48*	99.55*	99.55*	99.26*	99.31*
	TPA0910_00640	100	99.11	99.36	99.33	99.00	99.11
t1pks-6	TPA0910_41390	100	98.93	98.92	98.87	98.23	98.13
	TPA0910_41400	100	99.40	99.40	99.40	98.21	98.35
t2pks (spore pigment)	TPA0910_66950	100	99.37	99.37	99.37	98.82	98.66
	TPA0910_66940	100	98.80	98.72	98.72	98.96	98.96
	TPA0910_66930	100	100	99.61	100	99.22	99.22

pks/nrps-1 (alchivemycin)	TPA0910_49610	100	97.59	98.07	98.88	no	no
	TPA0910_49640	100	96.69	97.06	95.89	no	no
	TPA0910_49650	100	98.03	98.15	96.95	no	no
	TPA0910_49660	100	98.06*	97.01*	97.54	no	no
	TPA0910_49670	100	98.04*	98.06	97.81	no	no
	TPA0910_49680	100	98.84*	97.40	96.59	no	no
	TPA0910_49690	100	97.92*	97.66*	95.71	no	no
pks/mrps-2 (totopotensamide)	TPA0910_85480	100	99.72	99.63	99.81	no	no
	TPA0910_85540	100	99.74*	99.49*	99.17*	no	no
	TPA0910_85550	100	99.01*	99.45*	99.15*	no	no
	TPA0910_85650	100	99.36*	99.08	99.43	no	no
	TPA0910_85660	100	99.55*	99.61*	99.25*	no	no
	TPA0910_85750	100	99.57	99.51	99.39	no	no
pks/nrps-3	TPA0910_65820	100	99.76	99.68	99.68	no	no
	TPA0910_65840	100	99.68	99.52	99.42	no	no
	TPA0910_65870	100	99.38	99.39	99.60	no	no
	TPA0910_65880	100	99.50	99.52	99.55	no	no
pks/nrps-4	TPA0910_77610	100	99.60	99.34	99.74	99.47	99.08
	TPA0910_77590	100	98.97	98.84	99.23	98.20	98.33
	TPA0910_77530	100	99.37	99.32	99.18	99.07	99.15
	TPA0910_77520	100	99.65	99.86	99.72	99.31	99.17
	TPA0910_77490	100	97.94	97.81*	98.06	97.08*	97.47*

no, not observed; * not completely sequenced (query coverage is <90% in BLAST search)
